# Impact of valvular heart disease on activities of daily living of nonagenarians: the leiden 85-plus study a population based study

**DOI:** 10.1186/1471-2318-10-17

**Published:** 2010-04-01

**Authors:** Thomas van Bemmel, Victoria Delgado, Jeroen J Bax, Jacobijn Gussekloo, Gerard J Blauw, Rudi G Westendorp, Eduard R Holman

**Affiliations:** 1 Departments of Gerontology and Geriatrics, Leiden University Medical Center, PO Box 9600, 2300 RC Leiden, The Netherlands; 2 Department of Cardiology, Leiden University Medical Center, PO Box 9600, 2300 RC Leiden, The Netherlands; 3 Departments of Public health and Primary Care, Leiden University Medical Center, PO Box 9600, 2300 RC Leiden, The Netherlands

## Abstract

**Background:**

Data on the prevalence of valvular heart disease in very old individuals are scarce and based mostly on in-hospital series. In addition, the potential detrimental effect of valvular heart disease on the activities of daily living is unknown. The present study evaluated the prevalence of significant valvular heart disease and the impact of valvular heart disease on the activities of daily living in community dwelling nonagenarians. Nested within the Leiden 85-plus study, a population based follow-up study of the oldest old, a sample of 81 nonagenarians was recruited.

**Methods:**

The left ventricular (LV) dimensions, function and the presence and severity of heart valvular disease were evaluated by echocardiography. Significant valvular heart disease included any mitral or aortic stenosis severity, moderate or severe mitral regurgitation, moderate or severe aortic regurgitation and moderate or severe tricuspid regurgitation. Activities of daily living were assessed using the Groningen Activity Restriction Scale (GARS).

**Results:**

LV cavity diameters (end-diastolic diameter 47 ± 8 mm, end-systolic diameter 30 ± 8 mm) and systolic LV function (LV ejection fraction 66 ± 13%) were within normal for the majority of the participants. Significant valvular disease was present in 57 (70%) individuals, with mitral regurgitation and aortic regurgitation as the most frequent valve diseases (49% and 28% respectively). The GARS score between individuals with and without significant valvular heart disease was similar (36.2 ± 9.2 vs. 34.4 ± 13.2, p = 0.5).

**Conclusions:**

Nonagenarian, outpatient individuals have a high prevalence of significant valvular heart disease. However, no relation was observed between the presence of significant valvular heart disease and the ability to perform activities of daily living.

## Background

The increasing life expectancy of the population in the Western countries has determined an increase of chronic diseases, with cardiovascular disease being one of the most prevalent pathologies[1]. The elderly individuals comprise a growing demographic subgroup characterized by a high frequency of cardiovascular risk factors and co-morbidities[2]. Furthermore, this subgroup of individuals is often excluded from randomized trials, resulting in important implications for clinical management[3,4]. Particularly, data on the prevalence of valvular heart disease in very old individuals are scarce and based mostly on in-hospital series, introducing an important selection bias [4-7]. In addition, the potential detrimental effect of valvular heart disease on the activities of daily living is unknown.

The aim of the present study was twofold. First, the prevalence of significant valvular heart disease was evaluated in a group of outpatient individuals aged 90 years. Second, the impact of significant valvular heart disease on the performance in different activities of daily living was studied. Valvular heart disease was defined according to the Euro Heart Survey on Valvular Heart Disease[4].

## Methods

### Study protocol

The Leiden 85-plus Study is a prospective population-based study of all 85-year old inhabitants of Leiden, The Netherlands. The study design and characteristics of the cohort were described in detail previously[8,9]. In short, between September 1997 and September 1999 all 705 members of the 1912 to 1914-birth cohort in the city of Leiden were asked to participate in the month after their 85^th ^birthday. There were no selection criteria related to health or demographic characteristics. Participants were followed until death or the age of 90 years. At baseline and yearly thereafter, 85-year old participants were visited at their place of residence. During these visits blood pressure was measured, a venous blood sample drawn, an electrocardiogram recorded and face-to-face interviews and performance tests conducted. At 90 years of age, the participants were invited for an echocardiographic examination. Participants gave informed consent and for individuals who were severely cognitively impaired, a guardian gave informed consent. The Medical Ethics Commission of Leiden University approved the study.

### Clinical evaluation

Clinical status was evaluated in all individuals using the Groningen Activity Restriction Scale (GARS), a one-dimensional questionnaire that assess the performance in basic and instrumental activities of daily living (ADL and IADL)[10]. The score ranges from 18 to 90, higher scores indicating increasing disability. Cognitive function was assessed by the Mini-Mental State Examination (MMSE), with scores ranging from 0 to 30 points (optimal)[10]. Depressive symptoms were measured in those with MMSE > 18 points, using the 15-item Geriatric Depression Scale (GDS-15), with scores ranging from 0 to 15 points (optimal)[10]. A GDS-15 score above 4 was considered to be poor. The cardiovascular history was recorded, including history of coronary artery disease (medical history of angina or myocardial infarction), bypass surgery, stroke, and peripheral vascular disease[9]. Heart failure was defined as a positive response of the general practitioner to a specific question.

Blood pressure was measured twice with an interval of two weeks, using a mercury sphygmomanometer, in seating position after at least five minutes rest without having performed vigorous exercise during the preceding 30 minutes. We used the mean of the assessed systolic values and diastolic values.

### Echocardiography

Transthoracic 2-dimensional echocardiography was performed in all individuals in the left lateral decubitus position. Images were obtained using a commercially available system equipped with a 3.5-MHz transducer (Vingmed system Vivid-5; General Electric-Vingmed, Milwaukee, WI, USA). Standard gray-scale and color Doppler images were acquired at a depth of 16 cm at the parasternal (standard long- and short-axis images) and apical views (2-, 4-chamber and apical long-axis images). Data were stored for further off-line analysis.

Left ventricular (LV) diameters, interventricular septal (end-diastolic) thickness and posterior wall (end-diastolic) thickness were measured from M-mode images obtained from the parasternal long-axis view, and LV ejection fraction was derived using the Teichholz formula[11]. Left ventricular mass was calculated by the cube formula, using the correction formula proposed by Devereux *et al*[12]. Left ventricular mass was indexed by the body surface area. According to previous criteria, LV hypertrophy was defined by a LV mass index > 110 g/m^2 ^in women and >134 g/m^2 ^in men[13]. Finally, left atrial dimension was calculated by measuring the anteroposterior diameter from M-mode recordings of the parasternal long-axis view.

Left ventricular diastolic function was evaluated by measuring the transmitral peak velocities (E-wave and A-wave) obtained from pulsed-wave Doppler recordings of the transmitral inflow velocity; from these velocities the E/A ratio derived as another marker of LV diastolic function[13].

The valvular assessment included the evaluation of the function of the mitral, aortic and tricuspid valves. Color-Doppler echocardiography was performed after optimizing gain and Nyquist limit, and standard continuous and pulsed-wave Doppler recordings were acquired. Stenotic and regurgitant valve diseases were evaluated according to semiquantitative and quantitative methods recommended by the American Society of Echocardiography[13,14]. The severity of valvular stenosis was based on the valve area and the mean pressure gradient across the restrictive orifice[15]. The mitral valve area was calculated by the pressure half-time and the aortic valve area was calculated by the continuity equation[13,16]. The mean pressure gradient across the restrictive orifice was estimated by averaging the instantaneous gradients obtained from continuous wave Doppler recordings[13]. In addition, the severity of valvular regurgitation was determined on a qualitative scale and classified as mild (grade 1), moderate (grade 2) and severe (grades 3-4), according to the current ACC/AHA guidelines for the management of individuals with valvular heart disease[15]. According to the Euro Heart Survey on Valvular Heart Disease, significant valvular disease was defined as any mitral or aortic stenosis severity, moderate or severe mitral regurgitation, moderate or severe aortic regurgitation[4]. In addition, moderate or severe tricuspid regurgitation was considered as significant valvular heart disease. Finally, when tricuspid regurgitation was present, the pulmonary artery pressure was estimated using the modified Bernoulli equation.

### Statistical analysis

Continuous variables are presented as mean ± SD and categorical variables are presented as numbers and percentages. Differences between individuals with significant valvular disease and individuals without valvular disease were compared by the 2-tailed Student t-test, ANOVA, Mann-Whitney test and χ^2^-test for unpaired data, when appropriate. All statistical analyses were performed with software SPSS for Windows version 12.0 (SPSS, Inc., Chicago, IL). A p value <0.05 was considered statistically significant.

## Results

Of the initial 705 eligible participants at the Leiden 85-plus study, 14 died before they could be enrolled and 92 refused to participate, resulting in a cohort of 599 participants who could be enrolled at age 85 (87% response). Some 277 participants survived up to age 90 years and were in principle eligible for the study. Among them, 81 outpatient individuals underwent echocardiography. The remaining 196 individuals were not able to visit the study center. Clinical and demographic characteristics are presented in Table 1. All individuals with echocardiographic examination were 90 years old (33% men). The majority of the individuals were in sinus rhythm (93%). The mean body surface area was 1,7 m^2 ^[SD ± 0.7].

**Table 1:** Clinical and demographic characteristics

	Echocardiograpic examination		
	
	Yes [n = 81]	No [n = 196]	p value
Females [%]	67	75	0.20*
Living independently [%]	78	55	<0.001*
ADL (IQR) [points]	10 (9-14)	16 (10-25)	<0.001‡
IADL (IQR) [points]	23 (17-29)	33 (25-36)	<0.001‡
GARS (IQR) [points]	33 (26-42)	49 (35-61)	<0.001‡
MMSE (IQR) [points]	28 (26-29)	22 (15-27)	<0.001‡
GDS-15 (>4) [%]	14.8	32	<0.001‡
History of cardiovascular disease [%]†	35	43	0.15*
History of heart failure [%]	19	17	0.82*
Diastolic blood pressure (SD) [mmHg]	73 (9)	71 (10)	0.17*
Systolic blood pressure (SD) [mmHg]	155 (16)	150 (19)	0.03*

SD, Standard Deviation. IQR, Inter Quartile Range. ADL, Activities in Daily Living. IADL, Instrumental Activities in Daily Living. GARS, Groningen Activity Restriction Scale. MMSE, Mini Mental State Examination. GDS-15, Geriatric Depression Scale. †, Including cerebrovascular accident, angina pectoris, myocardial infarction, peripheral vascular disease or an electrocardiogram revealing myocardial ischemia or infarction. *, Independent T-test. ‡, Mann Whitney test. N, number of participants.

### Echocardiography

The echocardiographic characteristics of the study population are summarized in Table 2. The majority of the individuals showed normal LV dimensions and preserved systolic LV function. Seven (9%) individuals had LV systolic dysfunction (LV ejection fraction <50%). According to the previous definition, LV hypertrophy was present in 40 (51%) individuals, without differences between men and women (12 (44%) vs. 28 (55%), respectively; p = 0.5). Mean anteroposterior diameter of the left atrium was 42 ± 9 mm. An enlarged left atrium (anteroposterior diameter > 40 mm) was present in 43 (53%) individuals. Evaluation of LV diastolic function demonstrated impaired LV relaxation in the majority of the individuals, reflected by an E/A ratio <1.

**Table 2:** Left ventricular dimensions and function

	Mean [SD]
LV end-diastolic diameter (mm)	47 ± 8
LV end-systolic diameter (mm)	30 ± 8
Interventricular septum thickness (mm)	13 ± 3
Posterior wall thickness (mm)	11 ± 2
LV ejection fraction (%)	66 ± 13
LV mass index (g/m^2^)	126 ± 37
Left atrium diameter (mm)	42 ± 9
E/A ratio	0.7 ± 0.2

81 participants included. LV = left ventricular. SD = Standard Deviation.

Data on heart valve study are presented in Table 3. Significant valvular disease was observed in 57 (70%) individuals. Significant left-sided valvular disease involving only one valve (mitral or aortic) was noted in 38 (47%) individuals, whereas 23 (28%) individuals had both the mitral and the aortic valves involved.

**Table 3:** Valvular heart disease in the study population

Mitral Valve	
Mitral stenosis (%)	0 (0%)
Mitral regurgitation (%)	59 (73%)
Mild	20 (25%)
Moderate	24 (30%)
Severe	15 (19%)
**Aortic Valve**	
Aortic stenosis (%)	14 (17%)
Mild (mean ΔP < 25 mmHg)	9 (11%)
Moderate (mean ΔP 25 - 40 mmHg)	4 (5%)
Severe (mean ΔP > 40 mmHg)	1 (1%)
Aortic regurgitation (%)	38 (47%)
Mild	15 (19%)
Moderate	17 (21%)
Severe	6 (7%)
**Tricuspid valve**	
Tricuspid stenosis (%)	0 (0%)
Tricuspid regurgitation (%)	25 (31%)
Mild	8 (10%)
Moderate	5 (6%)
Severe	12 (15%)

81 participants included. ΔP = pressure gradient.

**Figure 1:**
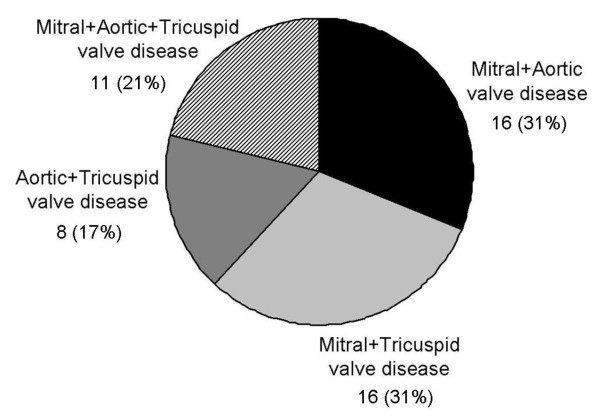
**Prevalence of combined significant valvular heart disease**.

Mitral and aortic regurgitation were the most common valvular diseases diagnosed and no patient had mitral stenosis. Significant mitral regurgitation was observed in 39 (49%) individuals: 24 (30%) individuals had moderate mitral regurgitation and 15 (19%) individuals had severe mitral regurgitation. Significant aortic regurgitation was observed in 23 (28%) individuals: 17 (21%) individuals had moderate aortic regurgitation and 6 (7%) individuals had severe aortic regurgitation. Aortic stenosis was present in 14 (17%) individuals: 9 (11%) individuals had mild aortic stenosis, 4 (5%) individuals had moderate aortic stenosis and only 1 (1%) patient had severe aortic stenosis. Finally, moderate to severe tricuspid regurgitation was observed in 17 (21%) individuals, together with significant left-sided valvular heart disease. The mean (SD) pulmonary artery pressure in these individuals was 35 (± 7) mmHg. Figure 1 shows the prevalence of combined significant valvular heart disease. Concomitant significant mitral valve disease and significant aortic or tricuspid valve disease were the most common combinations of valve disease.

### Ability to perform activities of daily living and heart valvular disease

Differences in functional status as assessed by the GARS score were evaluated between individuals with and without significant valve disease. Patients with and without moderate or severe valvular heart disease showed comparable GARS score: 34.8 ± 13.4 vs. 34.8 ± 9.4, p = 0.97. Furthermore, there were no differences in the GARS score between individuals without, one or two valves being significantly diseased (p = 0.6) (Table 4).

**Table 4:** Relation between GARS score and significant valvular disease

	No valvular disease (N = 20)	Any valvular disease (N = 61)	One valve diseased (N = 38)	Two valve diseased (N = 23)
GARS score				
Mean (SD)	36.2 ± 9.2	34.4 ± 13.2 *	35.4 ± 14.4	32.7 ± 11.1^†‡^

N = number of participants. * p = 0.5 vs. No valvular heart disease. † p = 0.4 one valve diseased vs. two valves diseased. ^‡ ^ANOVA p-value = 0.6 No valvular disease vs. one and two valves diseased. SD = Standard Deviation.

## Discussion

The findings in the current study illustrate that the majority of the nonagenarian outpatient individuals have normal LV cavity dimensions and normal systolic LV function on echocardiography. However, the prevalence of significant valvular disease is high, with mitral and aortic valve regurgitation being the most frequent valvular diseases. Nonetheless, the presence of significant valvular disease does not have a negative impact on the functional status that was assessed with the GARS score.

### Echocardiographic characteristics of the nonagenarian population

The nonagenarian outpatient population evaluated in the present study showed normal LV cavity dimensions and preserved systolic LV function. In contrast, diastolic LV function was characterized mostly by impaired LV relaxation. Several previous studies have reported similar findings in elderly individuals, with normal values of LV diameters and preserved systolic LV function, but with different grades of diastolic dysfunction[6,17]. As previously described, the aged-related changes in cardiac structure comprise a progressive increase in LV wall thickness whereas LV diameters and systolic LV function remain unchanged[18]. In addition, the increasing LV stiffness and collagen deposition that accompany the patient's aging, result in delayed LV relaxation and impairment of the early LV diastolic filling[18]. All these changes were observed in the present study, with an increased LV mass index (126 ± 37 g/m^2^) together with normal LV cavity diameters. Furthermore, various indices of diastolic dysfunction were abnormal (e.g. an E/A ratio <1 or an enlarged left atrium) indicating impaired LV compliance.

However, all these structural and functional changes are strongly influenced by the presence of cardiovascular disease (i.e. coronary artery disease, valvulopathies or hypertension), the most common co-morbidity among the elderly individuals.

Importantly, a high prevalence of valvular heart disease was observed in the present study population, with mitral and aortic valve regurgitation as the most frequent valvulopathies. Several contemporary series from Europe and the United States have demonstrated the relation between aging and the prevalence of degenerative valvular heart disease[4,6,7,19]. However, the true burden of valve disease remains still unclear since the majority of those series included in-hospital individuals or individuals referred for valvular surgery, introducing an important selection bias[4,6,7]. Recently, Nkomo et al. demonstrated, in a community study including 3851 individuals older than 75 years, high absolute rates of significant valvular heart disease (11.7%), with mitral regurgitation and aortic stenosis being the most frequent valve diseases (7.1% and 4.6%, respectively)[7]. Furthermore, Sadiq et al. evaluated the distribution pattern of moderate to severe valve disease among 63 hospitalised centenarian individuals, a significant minority of them were admitted because of cardiovascular events (35%)[6]. In that study, aortic stenosis was the most prevalent valve disease (27%) followed by mitral regurgitation (22%) and aortic regurgitation (17%)[6]. Whereas in the aforementioned series the population comprised individuals with known valve disease or in-hospital individuals,[6,7] the present population consisted of clinically stable and mostly independently living nonagenarians from the general population. The high prevalence of valvular heart disease as observed in the present study when compared to previous studies is difficult to explain because of the different clinical characteristics. As previously described, aortic stenosis is characterized by poor clinical tolerance and, therefore, may determine higher hospital admission rates and higher prevalence of this particular valve disease in the inpatient-based series.

### Impact of valvular disease on the ability in activities of daily living

In the current study, the competence in basic and instrumental activities of daily living was evaluated using the GARS score and related to the presence of valvular heart disease. Importantly, no differences in GARS score were observed between individuals with and without significant valve disease.

The relation between the impairments of basic activities of daily living and instrumental activities of daily living and cardiovascular co-morbidities has not been extensively studied[20,21]. Maugeri et al. related several parameters (e.g. walking speed, cognitive function, personal care) to systolic LV function in 170 individuals older than 70 years[21]. The authors demonstrated significant functional disability in those individuals with heart failure, whereas individuals with preserved systolic LV function obtained better scores in the tests[21]. In contrast, Formiga et al. evaluated the predictors of functional decline in 97 nonagenarians at 1 year follow-up and identified a history of stroke as an independent risk factor whereas the history of ischemic cardiomyopathy or heart failure were not related to functional decline[20].

The current study demonstrated that no relation existed between the performance in activities of daily living and the presence of significant valvular heart disease. Of note, the preserved systolic LV function observed in the majority of the population could preclude us to find differences in functional status between individuals with and without significant valve disease. Additional large studies, including individuals with a broad range of systolic LV function, may provide more insight into the relation between the competence in activities of daily living and the presence of valvular heart disease.

### Study Limitations

Although this was a population-based study, the included participants had an overall significant better physical and cognitive performance than those participants who were not included in this study. Therefore this study population represents a selected group of outpatient nonagenarians and hence, the results may not apply to the general population of nonagenarians.

Although, the GARS score is a validated score reflecting the limitations in daily life other more physical tests might have been more appropriate to use. However, the GARS score gives a valuable insight in the limitations of daily life that was related to valve dysfunction. That there is no apparent difference in those with versus those without significant valve dysfunction is therefore remarkable.

## Conclusions

The nonagenarian population has a high prevalence of significant valvular heart disease, in the presence of preserved systolic LV function, without LV dilatation. The presence of significant valve disease did not impact negatively on the ability to perform activities of daily living. Therefore, in this age group one should be cautious before undertaking any medical intervention because of valvulopathy.

## Competing interests

The authors declare that they have no competing interests.

## Authors' contributions

All authors participated in the design of the study and the analysis and interpretation of the data. JB, JG, GB and RW participated in the conception of the study. JB, JG, GB, RW and EH participated in the acquisition of data. All authors were involved with the draft and revisions of the manuscript. All authors have read and approved the final manuscript.

## Pre-publication history

The pre-publication history for this paper can be accessed here:

http://www.biomedcentral.com/1471-2318/10/17/prepub
